# Increased clonal hematopoiesis involving DNA damage response genes in patients undergoing lung transplantation

**DOI:** 10.1172/jci.insight.165609

**Published:** 2023-04-10

**Authors:** Laneshia K. Tague, Karolyn A. Oetjen, Anirudh Mahadev, Matthew J. Walter, Hephzibah Anthony, Daniel Kreisel, Daniel C. Link, Andrew E. Gelman

**Affiliations:** 1 Division of Pulmonary & Critical Care Medicine and; 2 Division of Oncology, Section of Stem Cell Biology, Department of Medicine, Washington University in St. Louis, St. Louis, Missouri, USA.; 3 UCLA, Los Angeles, California, USA.; 4 Department of Surgery, Division of Cardiothoracic Surgery, and; 5 Department of Pathology & Immunology, Washington University in St. Louis, St. Louis, Missouri, USA.

**Keywords:** Genetics, Transplantation, Genetic variation, Hematopoietic stem cells, Organ transplantation

## Abstract

**BACKGROUND:**

Cellular stressors influence the development of clonal hematopoiesis (CH). We hypothesized that environmental, inflammatory, and genotoxic stresses drive the emergence of CH in lung transplant recipients.

**METHODS:**

We performed a cross-sectional cohort study of 85 lung transplant recipients to characterize CH prevalence. We evaluated somatic variants using duplex error-corrected sequencing and germline variants using whole exome sequencing. We evaluated CH frequency and burden using χ^2^ and Poisson regression, and we evaluated associations with clinical and demographic variables and clinical outcomes using χ^2^, logistic regression, and Cox regression.

**RESULTS:**

CH in DNA damage response (DDR) genes *TP53*, *PPM1D*, and *ATM* was increased in transplant recipients compared with a control group of older adults (28% versus 0%, adjusted OR [aOR], 12.9 [1.7–100.3], *P* = 0.0002). Age (OR, 1.13 [1.03–1.25], *P* = 0.014) and smoking history (OR 4.25 [1.02–17.82], *P* = 0.048) were associated with DDR CH. Germline variants predisposing to idiopathic pulmonary fibrosis were identified but not associated with CH. DDR CH was associated with increased cytomegalovirus viremia versus patients with no (OR, 7.23 [1.95–26.8], *P* = 0.018) or non-DDR CH (OR, 7.64 [1.77–32.89], *P* = 0.024) and mycophenolate discontinuation (aOR, 3.8 [1.3–12.9], *P* = 0.031).

**CONCLUSION:**

CH in DDR genes is prevalent in lung transplant recipients and is associated with posttransplant outcomes including cytomegalovirus activation and mycophenolate intolerance.

**FUNDING:**

NIH/NHLBI K01HL155231 (LKT), R25HL105400 (LKT), Foundation for Barnes-Jewish Hospital (LKT), Evans MDS Center at Washington University (KAO, MJW), ASH Scholar Award (KAO), NIH K12CA167540 (KAO), NIH P01AI116501 (AEG, DK), NIH R01HL094601 (AEG), and NIH P01CA101937 (DCL).

## Graphical abstract

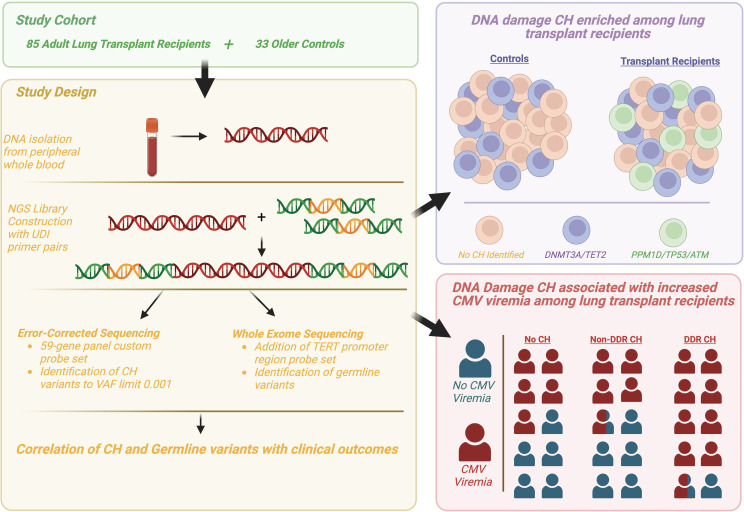

## Introduction

Clonal hematopoiesis (CH) is characterized by acquired somatic variants in hematopoietic cells that result in the increased fitness and clonal expansion of circulating leukocyte populations, rendering them detectable to genomic sequencing of peripheral blood. For certain leukemia-associated variants, CH has been studied as a precursor to hematologic malignancy; however, CH has also been associated with nonmalignant systemic manifestations, including atherosclerotic cardiovascular disease (1), thrombosis (2, 3), and sequela of congenital neutropenia syndromes (4). Most recently, an association of CH with severe infection and impaired inflammatory responses (5, 6), including in the setting of COVID-19 (5, 6), has led to interest in understanding the prevalence of CH in immunosuppressed populations. Among solid organ transplant recipients, patients undergoing lung transplantation experience the most intense immunosuppression regimens.

Selective immunologic and environmental pressures that contribute over time to clonal expansion of cells harboring somatic variants are beginning to be understood. Somatic variants in the epigenetic modifier genes *DNMT3A*, *TET2*, and *ASXL1* are the most common genes in CH and are associated with aging, inflammation, and tobacco exposure (7–14). Cytotoxic chemotherapy and radiation exposure are very strongly associated with CH in DNA damage response (DDR) genes, including *TP53*, *PPM1D*, *ATM*, and *CHEK2* (4, 15–19). As a result, cases of leukemia or myelodysplastic syndromes that arise following prior cancer treatment frequently have *TP53* or other DDR genes as driver mutations (17).

To gain additional insight into the pathogenesis of CH, we studied a cohort of patients with severe pulmonary disease requiring lung transplantation. The most common indications for lung transplantation are currently interstitial lung disease (ILD) and chronic obstructive pulmonary disease (COPD). This represents a shift toward an older lung transplant population in the sixth or seventh decade of life (20), and these patients have environmental, genetic, inflammatory, and medication exposures related to their diagnosis that create a unique combination of risk factors for CH. For COPD, an extensive history of tobacco use may precede lung transplantation; for idiopathic pulmonary fibrosis, underlying genetic predisposition for telomere biology disorders is increasingly recognized (21). Furthermore, undergoing lung transplantation necessitates lifelong posttransplant immunosuppression to maintain allograft function; this typically involves a combination regimen consisting of induction (basiliximab or rabbit antithymocyte globulin), calcineurin inhibitor, antiproliferative agent, and corticosteroids (20). The consequences of intense immunomodulation include alterations in T lymphocyte and B lymphocyte activation, proliferation, and effector functions; infection; posttransplant lymphoproliferative disease (22); other malignancies; and less-understood effects on granulocyte production and function (23–25). To varying degrees, immunosuppressive agents are postulated to exert cytotoxic stress, leading to cytopenias (26, 27). Of particular interest, the lymphocyte antiproliferative agent mycophenolate, an inosine monophosphate dehydrogenase inhibitor that blocks de novo guanosine synthesis, results in leukopenia in 23% of patients (28). Infection or severity of leukopenia due to mycophenolate requires use of alternative agents in many cases. We hypothesize that these risk factors and exposures contribute to increased CH within the population of lung transplantation recipients, and we sought to investigate patterns of CH variants and association with posttransplant outcomes.

In this study, we used a sensitive sequencing approach to characterize CH in 85 adult lung transplant recipients. We show that CH due to variants in DDR genes *TP53*, *PPM1D*, and *ATM* were markedly increased in lung transplant recipients compared with older control individuals. In contrast, the prevalence of CH due to common variants in epigenetic modifier genes *DNMT3A*, *TET2*, and *ASXL1* was not increased. In most cases, DDR CH was present prior to transplantation, suggesting that hematopoietic stressors present prior to transplantation selectively promote the expansion of hematopoietic clones carrying DDR gene variants. Finally, we show that DDR CH was associated with specific clinical outcomes, including CMV reactivation and lymphopenia.

## Results

### Clinical characteristics of lung transplant recipients.

The patient population included 85 adult lung transplant recipients, with clinical characteristics summarized in Table 1. The cohort of lung transplant recipients’ median age was 61 years, 60% were male, 91% identified as White, and 63% had a history of smoking. The most common indications for transplantation were ILD (52%) and COPD (29%). A control group was recruited from community centers and nursing homes; participants were at least 60 years of age with no acute illness, and this cohort was intended to be representative of a relatively healthy older adult population. The median age was 71 years — older than the cohort of transplant recipients (*P* < 0.0001) — and consisted of 73% women (*P* = 0.001) with 42% having history of smoking (*P* = 0.066).

### DDR CH is increased in lung transplant recipients.

**Figure 1:**
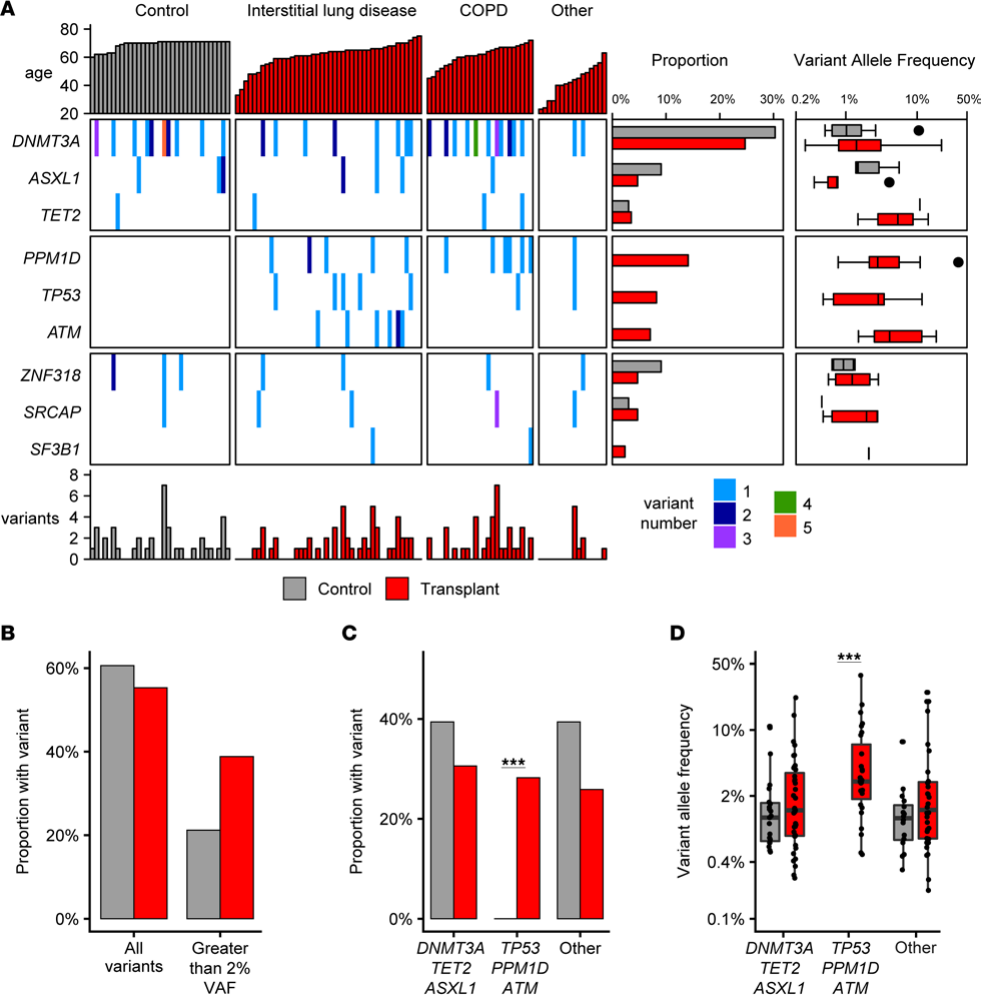
Clonal hematopoiesis in lung transplant recipients. (**A**) Somatic variants for lung transplant recipients (red, *n* = 85, grouped by transplant diagnosis) and older individuals (gray, *n* = 33) shown for top 9 genes. Total sum of all variants per individual indicated at bottom. For each gene, the proportion of older individuals and transplant recipients and variant allele frequencies are shown at right. (**B**) Proportion of transplant patients and older individuals with somatic variants detected with VAF > 0.1% (left) or > 2% (right). (**C**) Proportion of persons with a variant in *DNMT3A*, *TET2*, or *ASXL*; *TP53*, *PPM1D*, or *ATM*; or other genes. (**D**) VAF of somatic variants detected in *DNMT3A*, *TET2*, or *ASXL*; *TP53*, *PPM1D*, or *ATM*; or other genes. ***P < 0.001 assessed using Fisher’s exact test with adjustment for multiple comparisons. Box plot indicates median and first and third quartiles; whiskers indicate the range of all data points falling within 1.5 × interquartile range (IQR).

CH was assessed by genomic sequencing of peripheral blood samples. Error-corrected sequencing with duplex unique molecular identifiers was used to enable high-sensitivity somatic variant detection, with a variant allele frequency (VAF) threshold of 0.1% and robust discrimination from sequencing errors for a panel of 59 genes associated with CH or myeloid malignancies (Supplemental Table 1; supplemental material available online with this article; https://doi.org/10.1172/jci.insight.165609DS1). A total of 130 unique somatic variants were detected in 55% (*n* = 47 of 85) of lung transplant recipients and 61% (*n* = 20 of 33) of older individuals (Supplemental Table 2). Multiple variants were identified in 53% (*n* = 25 of 47) of lung transplant recipients (Figure 1A). The median VAF in lung transplant recipients was 1.35% (range, 0.16%–37.8%), and a VAF greater than 2% was detected in 39% (*n* = 33 of 85) of lung transplant recipients (Figure 1B). Within the older control group, CH with VAF greater than 2% was detected in 21% (*n* = 7 of 33, *P* > 0.1), and multiple variants were identified within the same individual in 35% (*n* = 7 of 20). No significant difference was observed in the proportion of persons with CH or in the median VAF in the 2 cohorts, including after correction of CH proportion for differences between these groups in age and sex (lung transplant recipients versus older controls [adjusted OR (aOR), 2.28 (0.82–6.35); *P* = 0.114]).

Strikingly, CH due to somatic variants in DDR genes, including *PPM1D*, *ATM*, or *TP53*, were identified exclusively in lung transplant recipients (Figure 1A). CH due to a DDR somatic variant was identified in 28% (*n* = 24 of 85) of lung transplant recipients (Figure 1C), and multiple DDR variants were identified in 3 lung transplant recipient specimens. CH due to DDR somatic variants was not detected in any older individuals, consistent with multiple large, published data sets (1, 9, 12), and was significantly different in lung transplant recipients [(aOR, 12.9 [1.7–100.3]; *P* = 0.0002). Analysis of the clinical characteristics of lung transplant patients with DDR CH are summarized in Supplemental Table 3. Age (aOR, 1.13 [1.03–1.25]; *P* = 0.014) and positive smoking history (aOR, 4.25 [1.02–17.82]; *P* = 0.048) were associated with DDR CH by multivariable analysis (Table 2). As reported in prior studies of elderly individuals, somatic variants in *DNMT3A*, *TET2*, or *ASXL1*, which are involved in epigenetic modification, were a common cause of CH, and their frequency was similar among lung transplant recipients and older controls (Figure 1D).

### DDR CH is present in patients with severe lung disease prior to transplantation.

**Figure 2:**
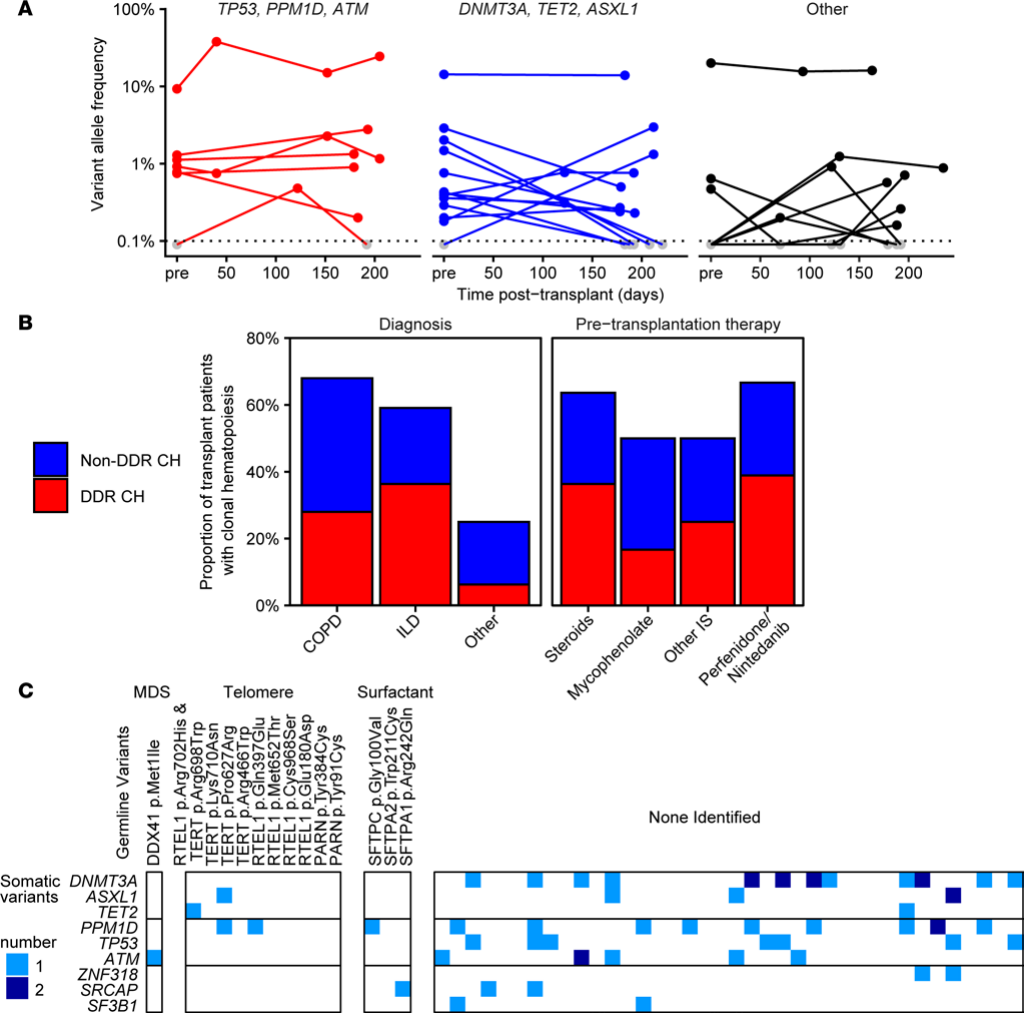
Clonal hematopoiesis is present in most patients prior to lung transplantation. (**A**) Longitudinal samples from a prospective cohort of patients were tracked pretransplant (pre) through posttransplant. Variant allele frequencies are shown on log scale for each somatic variant over time, grouped by *TP53*, *PPM1D*, and *ATM* (DDR, left, red) *DNMT3A*, *TET2*, and *ASXL1* (middle, blue), or other genes (right, black). (**B**) Proportion of lung transplantation recipients with any CH (top, blue) or DDR CH (bottom, red) based on lung disease diagnosis, pretransplantation immunosuppressive therapy, or presence of germline variant in surfactant or telomere maintenance genes. COPD, chronic obstructive lung disease; ILD, interstitial lung disease; IS, immunosuppressive therapy. (**C**) Whole exome sequencing in a subset of 52 lung recipients was interrogated for germline variants in genes associated with myeloid malignancies (*DDX41*), telomere biology disorders (*RTEL1*, *TERT*, *PARN*), or surfactant genes (*SFTPC*, *SFTPA1*, *SFTPA2*). Shown are pathogenic or uncertain significance germline variants (columns) versus somatic (clonal hematopoiesis) variants (rows).

A prospective subgroup of 20 lung transplant recipients enrolled prior to transplant had longitudinal samples available that included pretransplant and at least 1 posttransplant specimen. Like the cross-sectional posttransplant cohort, the prevalence of DDR CH in this subset was 25% (*n* = 5 of 20), including 1 patient harboring 2 DDR variants. Of the DDR variants detected at any time point, 83% (*n* = 5 of 6) of DDR variants could also be detected in specimens prior to transplantation (Figure 2A and Supplemental Table 2). Overall, CH was detectable prior to transplant for 65% of somatic variants identified in the prospective lung transplant cohort. The time between longitudinal samples spanned the most intense immunosuppression period during the first 6 months following transplant, and VAFs — including both DDR and non-DDR variants — were mostly stable over that time (Figure 2A). Patients who received treatment prior to transplant with steroids, mycophenolate, antifibrotic agents (pirfenidone or nintedanib), or other immunosuppression had similar prevalence of DDR CH compared with the overall cohort (Figure 2B).

### Germline predisposition to idiopathic pulmonary fibrosis and CH.

There is evidence that certain germline variants predispose to the development of CH. Genetic carriers of telomere biology disorders have an increased risk of developing both ILD and CH carrying DDR gene variants (29). To test the hypothesis that germline variants in genes predisposing in telomere maintenance genes are enriched in lung transplant patients with DDR CH, we performed whole exome sequencing on a subset of 52 patients, including all patients with ILD or DDR CH. We focused our analysis on telomere maintenance genes including *TERT*, *DKC1*, *TINF2*, *RTEL1*, and *PARN*, as well as other genes causing predisposition to pulmonary disease, bone marrow failure, or myeloid malignancies, as listed in Supplemental Table 4. Among patients with ILD, 27% (*n* = 12 of 44) of patients were identified with potential germline predisposition variants in telomere maintenance (*TERT*, *n* = 3; *RTEL1*, *n* = 5; *PARN*, *n* = 2; including 1 patient with both *RTEL1* and *TERT* variants) or surfactant genes (*SFTPA1*, *n* = 1; *SFTPA2*, *n* = 1; *SFTPC*, *n* = 1), all of which were heterozygous and putatively autosomal dominant (Supplemental Table 5). In addition, a potentially pathogenic variant in *TERT* was identified in a patient with COPD. Importantly, no increase in DDR CH was observed in patients with potentially pathogenic telomere maintenance genes (Figure 2C). DDR CH was present in 20% (*n* = 2 of 10) of patients with germline telomere gene variants and 33% (*n* = 1 of 3) of patients with surfactant germline variants (Figure 2B), and all 3 of these variants were in *PPM1D*.

### Cytomegalovirus (CMV) is activated at increased frequency in association with DDR CH.

**Figure 3:**
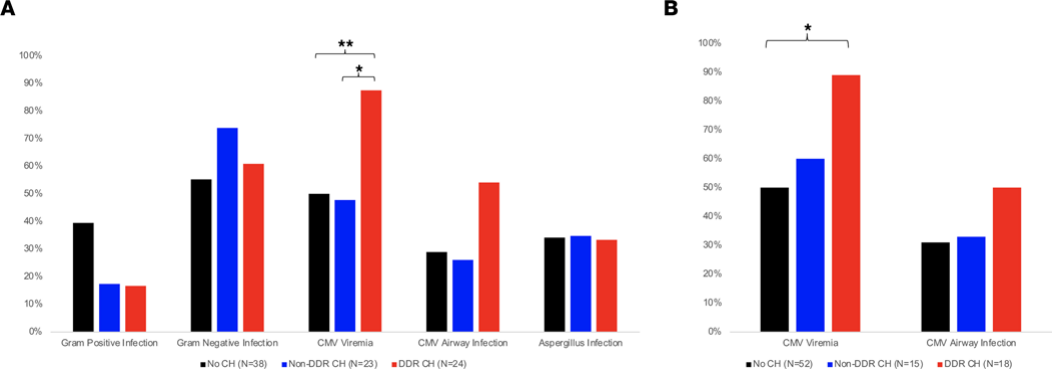
Association of clonal hematopoiesis with posttransplantation infection. (**A**) Proportion of lung transplant recipients with infectious complications following transplantation based on the presence of CH. (**B**) Proportion of lung transplant recipients with detectable CMV PCR in blood or airway following transplantation based on the presence of CH with a variant allele frequency of at least 2%. **P < 0.01, *P < 0.05 by χ^2^ with Bonferroni adjustment for multiple comparisons.

Recent descriptions of increased infection risk in patients with CH prompted analysis of infectious posttransplant complications and the association of overall and DDR-specific CH. No association of overall or DDR CH with gram-positive, gram-negative, or aspergillus infection was observed (Figure 3A). However, a striking association between CMV viremia and DDR CH was identified. CMV viremia was identified in 88% (*n* = 21 of 24) of patients with DDR CH, compared with 50% and 48% of patients with no CH or non-DDR CH, respectively (*P* = 0.018 and 0.024, respectively; Figure 3A). A nonsignificant increase in CMV^+^ respiratory PCR also was observed in patients with DDR CH (54.2% versus 26.1% in non-DDR CH and 28.9% in non-CH patients, *P* = 0.141). CMV viremia was also significantly associated with DDR CH when limited to VAF greater than 2% (*P* = 0.024, Figure 3B). Multivariable logistic regression analysis adjusted for age and CMV risk status showed a significant positive association between DDR CH and CMV viremia (Table 3; aOR, 7.71 [1.18–50.4]; *P* = 0.033).

### Hematologic abnormalities after lung transplantation and CH.

**Figure 4:**
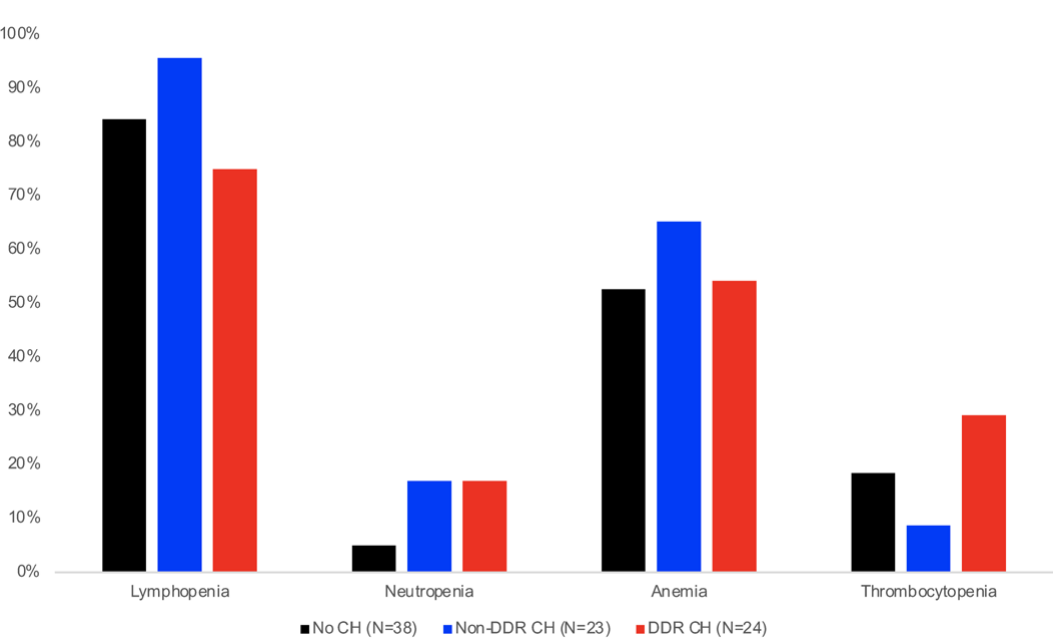
Association of clonal hematopoiesis with posttransplantation peripheral blood cytopenias. Proportion of lung transplantation recipients with no CH (*n* = 38), non-DDR CH (*n* = 23), or DDR CH (*n* = 24) with cytopenia. Lymphopenia, absolute lymphocyte count < 1,000 cells/μL; anemia, hemoglobin < 10.0 g/dL; thrombocytopenia, platelet count < 100,000 cells/μL; neutropenia, absolute neutrophil count < 1,000 cells/μL. *P <0.05 by χ^2^ with Bonferroni adjustment for multiple comparisons.

Cytopenias after lung transplantation are common and often provoked by immunosuppressive medications — in particular, mycophenolate. Neutropenia and lymphopenia increase risk for infection and are associated with reduced graft survival after lung transplantation (26). Lymphopenia (absolute lymphocyte count less than 1000 cells/μL) occurred in 84.7% (*n* = 72 of 85) of lung transplant recipients and was less likely to develop in patients with DDR CH, compared with non-DDR CH or no CH (75% versus 95.7% and 84.2%, respectively; *P* = 0.18; Figure 4). In multivariable Cox regression analysis adjusted for age, sex, transplant indication, CMV therapy, and MPA therapy, the presence of DDR CH was significantly associated with decreased lymphopenia (adjusted hazard ratio [aHR], 0.49 [0.27–0.90]; *P* = 0.021). The presence of neutropenia with absolute neutrophil count less than 1,000 cells/μL, which increases bacterial infection risk, occurred in 12% (*n* = 10 of 85) of lung transplant recipients but did not statistically differ between CH groups. Anemia or thrombocytopenia was not associated with CH. In accordance with standard institutional protocols, transplant recipients primarily received mycophenolate as initial antiproliferative immunosuppressive therapy; alternative treatment with azathioprine is most often considered when cytopenia or infection preclude ongoing treatment with mycophenolate. Interestingly, the presence of DDR CH was associated with discontinuation of mycophenolate, necessitating a switch to second line options [aOR 3.81 (1.13-12.86), *P* = 0.031, Supplemental Table 3].

Hematopoietic malignancies developed in 5 patients, including 1 myeloid malignancy and 4 lymphoid malignancies. Myelodysplastic syndrome occurred in a 73-year-old patient with ILD and no history of smoking. Four years to 6 years following lung transplant, serial bone marrow biopsy evaluations performed for neutropenia and macrocytic anemia diagnosed MDS with 10%–15% blasts. Sequencing of the peripheral blood at the time of MDS diagnosis identified a somatic pathogenic variant in *ATM* with VAF 18.5%, and exome sequencing in this study identified a germline pathogenic variant in *DDX41*. Lymphoid malignancies were identified in 4 cases: EBV^+^ posttransplantation lymphoproliferative disorder (*n* = 2), EBV^–^ diffuse large B cell lymphoma (*n* = 1), and chronic lymphocytic leukemia/small lymphocytic lymphoma (*n* = 1, concurrently diagnosed at the time of transplantation). No somatic or pathogenic germline variants were identified on sequencing in patients with lymphoid malignancies. Genes specifically reviewed in each exome sequencing result are listed in Supplemental Table 4.

## Discussion

CH has come to be understood as a consequence of aging and exposures such as tobacco, with the consequence of increased all-cause mortality even among individuals who never develop hematologic malignancy (2). Most remarkably, the present study finds that patients with severe lung disease undergoing lung transplantation have an increased prevalence of CH involving the DDR genes *PPM1D*, *ATM*, and *TP53* in 28% of cases. Consistent with multiple prior studies of older healthy individuals, which show that CH due to mutations of DDR genes is rare, we did not detect any DDR gene mutations in our control group (2, 12, 13). Within a prospective subgroup, pretransplant specimens could detect 83% of DDR variants that occurred after transplant, including all variants with VAF greater than 1%. This finding is unique to lung transplant recipients; recently, the reported incidence of CH in DDR genes (*TP53* and *PPM1D*) was only 4% in heart transplant recipients (30). DDR genes *TP53*, *ATM*, and *PPM1D* are known to have regulatory roles in cell division, survival, and tumor suppression (2). Unfortunately, utilizing a predesigned targeted gene panel limited the ability to further probe additional DDR genes of interest, such as *CHEK2*, after observing this strong association. To date, risk factors described for CH in DDR genes include chemotherapy and radiotherapy exposure (18), with particular variants at higher risk for driving therapy-related hematologic malignancy over the subsequent 10 years (17). Surprisingly, the case of myelodysplastic syndrome that occurred in a lung transplant recipient was found to have both *ATM* somatic variant and a pathogenic *DDX41* germline variant. Although *DDX41* has an established predisposition to myeloid malignancies, ILD has not been previously appreciated, and a larger patient cohort will be needed to establish whether there is an associated risk.

As expected, among lung transplant recipients, both age and smoking exposure were associated with the presence of CH. Common variants in *DNMT3A*, *ASXL1*, and *TET2* typically associated with aging and tobacco exposure were present in both transplant recipients and older individuals with similar prevalence. Of note, detection of *TET2* variants was lower in this study than historical cohorts due to lower probe set coverage; however, remaining genes were adequately covered for high sensitivity. For studies of malignant disease, CH with VAF greater than 2% has the strongest available evidence of association with future hematologic malignancies (31). In contrast, more sensitive methods identify smaller clonal expansions, which have the potential to expand further when the variant confers increased hematopoietic stem cell fitness (32). The threshold of VAF to define clinical impact is less well defined for nonmalignant systemic manifestations of CH, largely due to the lower sensitivity of sequencing approaches employed for population-based studies, and is also potentially variant specific (12, 13, 18). Since the lung transplant recipients in this study had not experienced pretransplant chemotherapy or radiotherapy, this patient population represents a potentially novel risk population for DDR CH.

Our data suggest that the majority of DDR CH is present prior to lung transplantation. This raised the possibility that germline variants that predispose to ILD and other pulmonary phenotypes may also predispose to the development of DDR CH. Telomere attrition is known to induce DDR pathways involving *ATM*, *PPM1D*, and *TP53* and, when critically shortened, can lead to proliferative arrest, particularly in T lymphocytes (33, 34). Telomere biology disorders have a reported increase in prevalence of CH due to variants in *PPM1D* (35). DDR CH was present at similar prevalence in patients both with and without germline variants commonly associated with telomere biology disorders. The absence of *TERC* detection in exome sequencing may have missed some cases, and additional inherited predisposition variants likely remain to be described. Telomere length was not directly measured in this study. Such analysis might identify additional cases with a telomere biology disorder, which would help clarify the relationship between telomere maintenance and development of CH in the lung transplant setting

We identified a significant association between DDR CH and CMV viremia following lung transplant, and this persisted even after controlling for CMV serostatus of recipient and donor. Notably, lung transplant recipients with idiopathic pulmonary fibrosis have decreased cytotoxic T cell proliferative responses in response to CMV antigen challenge (36). Additionally, CMV infection is known to accelerate immune aging and impair memory CD8^+^ T cell proliferation, signaling, and cytokine production (37). Indeed, immune age is positively associated with both increased risk of CMV infection and decreased survival (38). Genotoxic stress from telomere shortening has been implicated in both complications from antiproliferative therapy and increased CMV infection risk (36, 39–41). Although the prospective cohort in our data is too small to assess for causality, it raises the possibility that preexisting DDR CH results in T lymphocyte dysfunction and may lead to CMV-mediated allograft tissue injury and poor lung transplant outcomes. Studies of T cell subsets and assessment of CD8^+^ T cell function were not performed in this initial study but would provide further support of these observations in future specimens. Based on these findings, extended longitudinal studies are underway to assess for adverse clinical outcomes including acute cellular rejection, antibody-mediated rejection, chronic lung allograft dysfunction, and mortality in a larger prospective cohort of lung transplant recipients.

In conclusion, the prevalence of CH in patients with severe lung disease undergoing transplantation is remarkably high in DDR genes compared with older adults. We demonstrate that these DDR variants arise in patients with severe lung disease either with or without germline predisposition to telomere biology disorders, and they develop predominantly prior to transplantation. Over the most intense period of immunosuppression in the first 6 months following transplant, CH VAFs do not have patterns of consistent growth or regression. Outcomes of CMV activation in blood and bronchial fluid, as well as mycophenolate discontinuation, are observed in lung transplant patients with DDR CH.

## Methods

### Study population.

All participants were enrolled at Washington University in St. Louis. A prospective cross-sectional cohort study included 20 adult lung transplant patients with transplants between January and December 2020, with sequential peripheral whole-blood samples collected both immediately prior to transplantation and at 6 months after transplantation. Clinical outcomes for these patients were reviewed for a minimum follow-up period of 1 year. Single time point peripheral blood samples were collected from an additional 65 adult lung transplant recipients at a median of 4 years after transplant and from 33 deidentified control individuals. All demographic and clinical data for lung transplant patients was extracted from the electronic medical record. The primary outcome was overall CH prevalence. Secondary outcomes included CH variant rates and association of CH with infection and cytopenia after lung transplantation. Infection was defined as a positive respiratory culture (bronchial wash or lavage, or tracheal aspirate) for bacterial and fungal infections or as a positive respiratory or nasopharyngeal PCR test for viral infections. For CMV, peripheral blood was assessed by qualitative and quantitative PCR (qPCR) based on standard clinical posttransplant guidelines. For cytopenias, lymphopenia was defined as an absolute lymphocyte count < 1,000 cells/μL, neutropenia as an absolute neutrophile count < 1,000 cells/μL, anemia as hemoglobin < 10 g/dL, and thrombocytopenia as < 100,000 cells/μL.

### Clinical immunosuppression management.

All immunosuppression regimens were managed independently of the current study at the discretion of the treating clinical transplant pulmonologist. During the study period, the institutional protocol for immunosuppression for lung transplant recipients included induction with basiliximab or rabbit anti-thymocyte globulin and a maintenance regimen of 3 medications: calcineurin inhibitor, antiproliferative agent, and corticosteroids. Specifically, basiliximab was the first choice for immunosuppression induction; however, recipients with positive donor crossmatch at the time of transplant may have substituted rabbit antithymocyte globulin. The maintenance regimen consisted of tacrolimus with trough goal level 8–10 ng/mL for the first year following transplant, mycophenolate 1,000 mg twice daily, methylprednisolone 0.5 mg/kg twice daily for 6 doses, and prednisone 0.5 mg/kg/day starting at completion of methylprednisolone and tapering over 6 months following transplant to goal dose, at 5 mg daily. If clinically indicated, cyclosporine was used as an alternative for tacrolimus, and azathioprine was used as an alternative to mycophenolate.

### Error-corrected sequencing.

DNA was extracted from peripheral whole blood specimens (QIAamp DNA mini, QIAGEN) from lung transplant recipients and older adults. Archived DNA from 9 deidentified healthy pediatric peripheral blood samples was used as a technical control for variant calling and sequencing artifacts (4). Input of 200 ng DNA was used for library preparation with unique dual index primer pairs (xGen Prism DNA Library Prep Kit with xGen UDI Primer Pairs, IDT). Libraries were hybridized twice with a custom capture probe pool (IDT) of 59 genes (Supplemental Table 1) and were then amplified. Sequencing of captured libraries was performed using the NovaSeq S4 300/XP (Illumina) at the McDonnell Genome Institute of Washington University in St. Louis.

Demultiplexed fastq files were used to generate an unmapped bam for extraction of unique molecular index sequences (fgbio version 1.3.0; ExtractUmisFromBam, readstructure 8M143T 8M143T; ref. 42) and mapping to hg19 (picard and bwa mem, version 0.7.15-r1140; refs. 43 and 44) with UMI error correction (CorrectUmis, max-mismatches = 3, min-distance = 1). Reads were grouped by read families (GroupReadsByUmi, edits = 0, min-map-q = 20) then collapsed (CallDuplexConsensusReads, error-rate-pre-umi = 45, error-rate-post-umi = 30, min-input-base-quality = 30), realigned, filtered (FilterConsensusReads, min-reads = 2 1 1, max-read-error-rate = 0.05, max-base-error-rate = 0.1, min-base-quality = 50, max-no-call-fraction = 0.05) and hard clipped for overlap between read pairs (ClipBam).

Variant calls were produced using 3 variant callers in tumor only mode: VarDict Java 1.8.2 (45), Mutect2 (GATK, 4.2.2.0; ref. 46), and VarScan 2.4.2 (47), with minimum VAF 0.001. Variants were annotated with VEP version 104_GRCh37 (48). Variants were required to be called by at least 2 variant callers with VAF less than 40% to filter germline single-nucleotide polymorphisms. gnomAD population allele frequencies (49) and ClinVar (50) annotations were reviewed for variants with VAF greater than 30% to manually discriminate germline and somatic variants. In pediatric control samples, 13 genomic regions with identified variants were manually reviewed and excluded from all samples due to slippage and mapping artifacts in repetitive sequences. For samples obtained from serial time points, confirmed variants were also reviewed in all time points within single-read families (generated using CallMolecularConsensusReads parameters error-rate-pre-umi = 45, error-rate-post-umi = 30, min-input-base-quality = 30, min-reads = 1, min-consensus-base-quality = 40, and FilterConsensusReads parameters min-reads = 3, max-read-error-rate = 0.05, max-base-error-rate = 0.1, min-base-quality = 40, max-no-call-fraction = 0.1).

All variants identified in the analysis pipeline using allele frequency threshold greater than 0.1% were manually reviewed, visualized using Complex Heatmap (51), and included in statistical comparisons of outcomes. When noted in the text, a subset of variants with allele frequency greater than 2% were used for additional comparisons.

### Whole exome sequencing.

A total of 52 patients with diagnosis of ILD or with somatic variants detected in *ATM*, *PPM1D*, and *TP53* underwent additional exome sequencing. Previously prepared xGen Prism DNA libraries were hybridized with the xGen Lockdown Exome Panel v1 capture set with an additional probe set for TERT promoter region. Sequencing was performed using NovaSeq S4 300XP to goal 30× coverage at the McDonnell Genome Institute. Analysis was performed using DRAGEN for alignment to the hg38 reference genome and quality filtering. Variants were annotated using ANNOVAR and VEP for comparison to publicly available databases of somatic and germline variants (52).

### Statistics.

Demographic and clinical variables were analyzed with descriptive statistics. We evaluated continuous variables utilizing Pearson correlation or Wilcoxon test and categorical variables utilizing either Fisher’s exact or χ^2^ test as appropriate. We performed logistic regression to model the relationship between CH and clinical/demographic variables. Poisson regression was used to determine differences in CH variant burden (number of distinct CH variants identified per patient). We evaluated the relationship between CH and infectious and hematologic outcomes with the χ^2^ test and logistic regression. We adjusted for multiple comparisons using the Bonferroni method. For χ^2^ analyses of binary outcome, this including adjusting for 6 total tests (3 CH groups, 2 outcomes). We used multivariable regression modelling to assess the influence of age, sex, transplant diagnosis, and other appropriate outcome-specific variables identified based on known or purported confounding associations. For all analyses, a 2-sided *P* value of 0.05 was considered significant. All statistical analyses were performed with IBM SPSS Statistics Version 27 (IBM Corp.) and R version 4.0.2/R Studio version 1.3.1093 (53).

### Study approval.

This study was approved by the IRB at Washington University in St. Louis (IRB, 201911002). Written informed consent was obtained from all study participants prior to participation in the study.

## Author contributions

LKT contributed to designing the research study, acquiring data, analyzing the data, and writing the manuscript. KAO contributed to acquiring data, analyzing data, and writing the manuscript. AM contributed to acquiring data and writing the manuscript. HA contributed to acquiring and analyzing data and reviewed the manuscript. MJW contributed to acquiring and analyzing data and reviewed the manuscript. DCL contributed to designing the research study, acquiring data, analyzing the data, and writing the manuscript. AEG contributed to designing the research study, acquiring data, analyzing the data, and writing the manuscript. DK contributed to designing the research study and writing and reviewing the manuscript.

## Supplementary Material

Supplemental data

ICMJE disclosure forms

## Figures and Tables

**Table 1:**
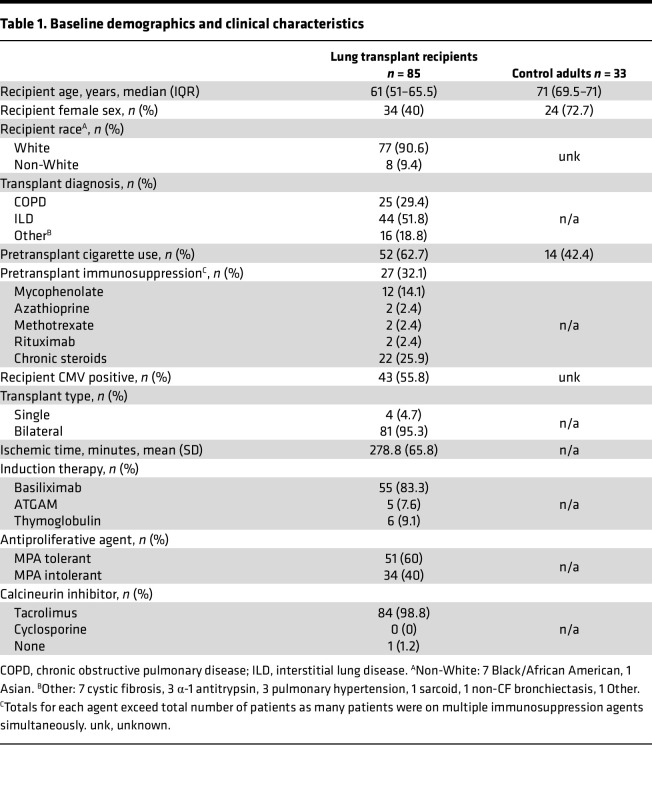
Baseline demographics and clinical characteristics

**Table 2:**
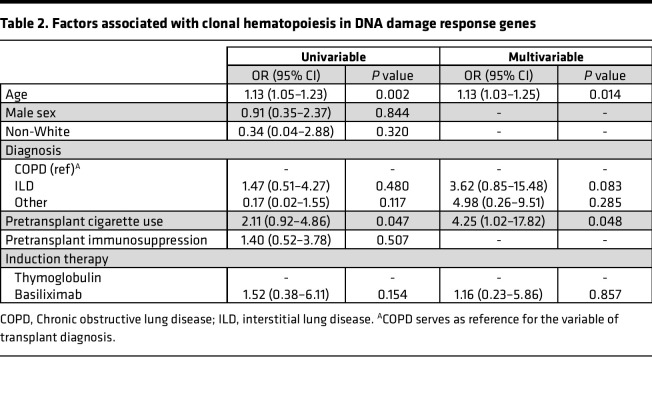
Factors associated with clonal hematopoiesis in DNA damage response genes

**Table 3:**
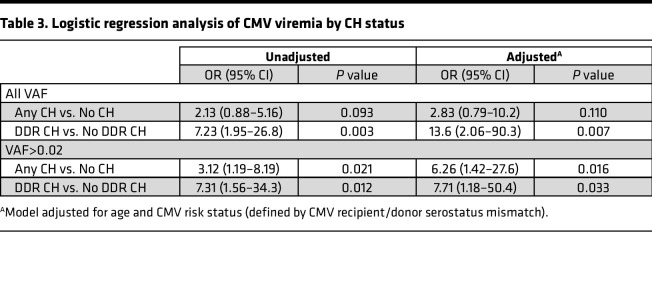
Logistic regression analysis of CMV viremia by CH status

## Version 1 02/28/2023

In-Press Preview

## Version 2 04/10/2023

Electronic publication
