# Supplementary material for: Increased clonal hematopoiesis involving DNA damage response genes in patients undergoing lung transplantation
Source: JCI Insight. 2023 Apr 10;8(7):e165609. doi: 10.1172/jci.insight.165609 (PMC10132147; doi:10.1172/jci.insight.165609)
Supplement: Supplemental data [file jciinsight-8-165609-s144.pdf]

Supplementary Materials

Table of Contents

Table S1: Genes included on Custom CH Gene Panel

Table S2: CH Variant Details

Table S3: Lung Transplant Recipient Clinical Characteristics by CH Status

Table S4: Genes Reviewed on Whole Exome Sequencing

Table S5: Potentially Pathogenic Germline Variants

Table S1: Genes Included on Custom CH Gene Panel

ASXL1	*ATM*	*ATRX*	*BCOR*	*BCORL1*	*BRCC3*
BUB1B	*CBL*	*CEBPA*	*CEBPB*	*CREBBP*	*CSF3R*
CUX1	*CXCL12*	*CXCR2*	*CXCR4*	*DNMT3A*	*EPO*
EZH2	*FLT3*	*GNAS*	*GNB1*	*HBB*	*IDH1*
IDH2	*IL17RA*	*JAK2*	*KDM6A*	*KRAS*	*LUC7L2*
MYD88	*NPM1*	*NRAS*	*PHF6*	*PKLR*	*PPM1D*
PTPN11	*RAD21*	*RUNX1*	*SETBP1*	*SETD2*	*SF1*
SF3B1	*SH2B3*	*SMARCD2*	*SMC1A*	*SMC3*	*SRCAP*
SRSF2	*STAG2*	*STAT3*	*TET2*	*TFR2*	*TP53*
TUBB1	*U2AF1*	*ZBTB33*	*ZNF318*	*ZRSR2*	

Table S2: CH Variant Details

UPN	Cohort	Gene	HGVSg	HGVSp	Consequence	VAF (%)
006-13	post-transplant	BCORL1	chrX:g.129159291A>G	ENSP00000437775.1:p.Lys1339Glu	missense_variant	0.7
006-13	post-transplant	ATM	chr11:g.108216546G>A	ENSP00000278616.4:p.Arg2832His	missense_variant	8.82
006-26	post-transplant	SRCAP	chr16:g.30718896del	ENSP00000262518.4:p.Val166CysfsTer4	frameshift_variant	2.75
006-30	post-transplant	SF3B1	chr2:g.198266834T>C	ENSP00000335321.5:p.Lys700Glu	missense_variant	2.09
006-30	post-transplant	KDM6A	chrX:g.44942752G>C	ENSP00000367203.4:p.Arg1111Pro	missense_variant	1.05
006-30	post-transplant	ZBTB33	chrX:g.119388871C>G	ENSP00000314153.2:p.Ala534Gly	missense_variant	15.83
006-30	post-transplant	PPM1D	chr17:g.58740525del	ENSP00000306682.2:p.Asn477IlefsTer6	frameshift_variant	11
006-30	post-transplant	SRSF2	chr17:g.74733073A>T	ENSP00000376276.2:p.Phe57Tyr	missense_variant	20
006-52	post-transplant	DNMT3A	chr2:g.25457266T>C	ENSP00000264709.3:p.Tyr874Cys	missense_variant	3.8
006-52	post-transplant	TP53	chr17:g.7577120C>T	ENSP00000269305.4:p.Arg273His	missense_variant	11.69
006-74	post-transplant	SRCAP	chr16:g.30740326C>T	ENSP00000262518.4:p.Arg1900Cys	missense_variant	2.5
007-02	post-transplant	DNMT3A	chr2:g.25470545A>G	ENSP00000264709.3:p.Ile310Thr	missense_variant	1.41
007-36	post-transplant	DNMT3A	chr2:g.25505528G>A	ENSP00000264709.3:p.Ser77Phe	missense_variant	0.6
007-36	post-transplant	SRCAP	chr16:g.30735967G>T	ENSP00000262518.4:p.Gly1741Val	missense_variant	0.48
007-36	post-transplant	TP53	chr17:g.7578401G>T	ENSP00000269305.4:p.Pro177Thr	missense_variant	0.5
007-36	post-transplant	STAT3	chr17:g.40474309C>A	ENSP00000264657.4:p.Asp698Tyr	missense_variant	1.33
007-36	post-transplant	PPM1D	chr17:g.58740525del	ENSP00000306682.2:p.Asn477IlefsTer6	frameshift_variant	1.61
007-48	post-transplant	TP53	chr17:g.7578479G>T	ENSP00000269305.4:p.Pro151Thr	missense_variant	2.84
008-07	post-transplant	DNMT3A	chr2:g.25467491C>T	ENSP00000264709.3:p.Asp529Asn	missense_variant	0.94
008-28	post-transplant	SMC1A	chrX:g.53432558A>G	ENSP00000323421.3:p.Leu593Pro	missense_variant	1.2
008-34	post-transplant	ATM	chr11:g.108117798C>T	ENSP00000278616.4:p.Arg337Cys	missense_variant	18.52
008-40	post-transplant	BCORL1	chrX:g.129189835C>A	ENSP00000437775.1:p.Cys1620Ter	stop_gained	3.59
008-40	post-transplant	PPM1D	chr17:g.58740383del	ENSP00000306682.2:p.Val430Ter	frameshift_variant	2.94
008-40	post-transplant	ASXL1	chr20:g.31024320del	ENSP00000364839.4:p.Ser1269ArgfsTer11	frameshift_variant	0.75
008-40	post-transplant	CEBPB	chr20:g.48808127G>T	ENSP00000305422.3:p.Arg186Leu	missense_variant	0.84
008-57	post-transplant	PPM1D	chr17:g.58740444del	ENSP00000306682.2:p.Leu450Ter	frameshift_variant	2.13
008-62	post-transplant	DNMT3A	chr2:g.25470022A>T	ENSP00000264709.3:p.Cys340Ter	stop_gained	3.23
008-62	post-transplant	SMC1A	chrX:g.53432009G>C	ENSP00000323421.3:p.Arg711Gly	missense_variant	7.04
008-62	post-transplant	ATM	chr11:g.108121531C>T	ENSP00000278616.4:p.Arg447Ter	stop_gained	15.32
008-62	post-transplant	ATM	chr11:g.108224595G>C	ENSP00000278616.4:p.Gly2925Ala	missense_variant	4.11
008-77	post-transplant	DNMT3A	chr2:g.25464544C>T	ENSP00000264709.3:p.Val657Met	missense_variant	7.52
008-77	post-transplant	ATM	chr11:g.108196887G>C	ENSP00000278616.4:p.Glu2304Gln	missense_variant	1.5
008-77	post-transplant	ASXL1	chr20:g.31021439G>T	ENSP00000364839.4:p.Glu480Ter	stop_gained	4.07
009-40	post-transplant	DNMT3A	chr2:g.25462022C>A	ENSP00000264709.3:p.Trp795Cys	missense_variant	1.45
009-40	post-transplant	DNMT3A	chr2:g.25464568C>A	ENSP00000264709.3:p.Val649Leu	missense_variant	1.02

Table S2: CH Variant details (continued)

UPN	Cohort	Gene	HGVSg	HGVSp	Consequence	VAF (%)
009-40	post-transplant	DNMT3A	chr2:g.25469028C>T	NA	splice_donor_variant	0.49
009-40	post-transplant	DNMT3A	chr2:g.25469489C>A	ENSP00000264709.3:p.Glu427Ter	stop_gained&splice_region_variant	2.35
009-45	post-transplant	SF3B1	chr2:g.198266611C>T	ENSP00000335321.5:p.Gly742Asp	missense_variant&splice_region_variant	2.11
009-45	post-transplant	PPM1D	chr17:g.58740633T>A	ENSP00000306682.2:p.Leu513Ter	stop_gained	9.43
010-03	post-transplant	DNMT3A	chr2:g.25469129del	ENSP00000264709.3:p.Glu444ArgfsTer207	frameshift_variant	2.73
010-03	post-transplant	ZNF318	chr6:g.43325336del	ENSP00000354964.2:p.His239LeufsTer42	frameshift_variant	1.65
010-25	post-transplant	RUNX1	chr21:g.36252869C>T	ENSP00000300305.3:p.Gly165Ser	missense_variant	6.01
010-50	post-transplant	PPM1D	chr17:g.58740502del	ENSP00000306682.2:p.Asp470IlefsTer13	frameshift_variant	2.8
011-42	post-transplant	ATM	chr11:g.108199871A>C	ENSP00000278616.4:p.Met2405Leu	missense_variant	2.3
011-42	post-transplant	ASXL1	chr20:g.31024609G>T	ENSP00000364839.4:p.Gly1365Val	missense_variant	0.56
011-76	post-transplant	DNMT3A	chr2:g.25466787A>C	ENSP00000264709.3:p.Leu639Arg	missense_variant	21.97
011-76	post-transplant	DNMT3A	chr2:g.25471070G>A	ENSP00000264709.3:p.Gln231Ter	stop_gained	0.95
011-76	post-transplant	PPM1D	chr17:g.58740756dup	ENSP00000306682.2:p.Ser556LysfsTer4	frameshift_variant	5.59
013-23	post-transplant	TP53	chr17:g.7577538C>T	ENSP00000269305.4:p.Arg248Gln	missense_variant	3.12
013-47	post-transplant	DNMT3A	chr2:g.25457243G>A	ENSP00000264709.3:p.Arg882Cys	missense_variant	6.91
013-47	post-transplant	DNMT3A	chr2:g.25470464G>A	ENSP00000264709.3:p.Ser337Leu	missense_variant	4.2
013-47	post-transplant	DNMT3A	chr2:g.25470533C>T	ENSP00000264709.3:p.Trp314Ter	stop_gained	0.99
013-47	post-transplant	CXCL12	chr10:g.44873264C>T	ENSP00000379140.2:p.Arg119Gln	missense_variant	1.94
013-47	post-transplant	SRCAP	chr16:g.30723707T>G	ENSP00000262518.4:p.Leu647Arg	missense_variant	1.52
013-47	post-transplant	SRCAP	chr16:g.30724931del	ENSP00000262518.4:p.Gln798SerfsTer14	frameshift_variant	2.79
013-47	post-transplant	SRCAP	chr16:g.30744990G>A	ENSP00000262518.4:p.Gly2122Asp	missense_variant	0.64
013-50	post-transplant	DNMT3A	chr2:g.25463289T>C	ENSP00000264709.3:p.Tyr735Cys	missense_variant	1.9
013-50	post-transplant	DNMT3A	chr2:g.25470535C>T	ENSP00000264709.3:p.Trp313Ter	stop_gained	1.36
013-50	post-transplant	TP53	chr17:g.7577568C>T	ENSP00000269305.4:p.Cys238Tyr	missense_variant	3.8
013-57	post-transplant	ATM	chr11:g.108139175C>T	ENSP00000278616.4:p.Gln893Ter	stop_gained	2.74
013-58	post-transplant	DNMT3A	chr2:g.25463578C>A	ENSP00000264709.3:p.Asp702Tyr	missense_variant	3.96
013-58	post-transplant	DNMT3A	chr2:g.25467204G>T	ENSP00000264709.3:p.Cys557Ter	stop_gained	5.41
013-58	post-transplant	PPM1D	chr17:g.58740533_58740534insT	ENSP00000306682.2:p.Lys480IlefsTer9	frameshift_variant	3.7
013-59	post-transplant	CREBBP	chr16:g.3781420T>G	ENSP00000262367.5:p.Ile1649Leu	missense_variant	25
013-60	post-transplant	DNMT3A	chr2:g.25457185A>C	ENSP00000264709.3:p.Leu901Arg	missense_variant	0.83
013-65	post-transplant	TET2	chr4:g.106156036dup	ENSP00000442788.1:p.Thr313AsnfsTer18	frameshift_variant	5.36
013-67	post-transplant	DNMT3A	chr2:g.25469508del	ENSP00000264709.3:p.Gly421AlafsTer230	frameshift_variant	0.75
013-67	post-transplant	ZNF318	chr6:g.43320216T>C	NA	splice_acceptor_variant	2.86
015-01	pre-transplant	DNMT3A	chr2:g.25470459C>T	NA	splice_donor_variant	0.43
015-01	pre-transplant	TET2	chr4:g.106164778C>T	ENSP00000442788.1:p.Arg1216Ter	stop_gained	14.29
015-01	pre-transplant	PPM1D	chr17:g.58740483del	ENSP00000306682.2:p.Gly463ValfsTer2	frameshift_variant	0.78

Table S2: CH Variant details (continued)

UPN	Cohort	Gene	HGVSg	HGVSp	Consequence	VAF (%)
015-01	post-transplant	TET2	chr4:g.106164778C>T	ENSP00000442788.1:p.Arg1216Ter	stop_gained	13.89
015-01	post-transplant	PPM1D	chr17:g.58740483del	ENSP00000306682.2:p.Gly463ValfsTer2	frameshift_variant	0.2
015-04	post-transplant	DNMT3A	chr2:g.25463239A>C	ENSP00000264709.3:p.Phe752Val	missense_variant	1.32
015-04	post-transplant	DNMT3A	chr2:g.25463299A>C	ENSP00000264709.3:p.Phe732Val	missense_variant	2.97
015-04	post-transplant	DNMT3A	chr2:g.25463239A>C	ENSP00000264709.3:p.Phe752Val	missense_variant	0.3
015-04	post-transplant	DNMT3A	chr2:g.25463299A>C	ENSP00000264709.3:p.Phe732Val	missense_variant	2.61
015-04	pre-transplant	DNMT3A	chr2:g.25463299A>C	ENSP00000264709.3:p.Phe732Val	missense_variant	0.18
015-05	post-transplant	STAT3	chr17:g.40474482T>A	ENSP00000264657.4:p.Tyr640Phe	missense_variant	0.88
015-05	post-transplant	STAT3	chr17:g.40474482T>A	ENSP00000264657.4:p.Tyr640Phe	missense_variant	1.24
015-07	post-transplant	DNMT3A	chr2:g.25464478C>T	ENSP00000264709.3:p.Gly679Arg	missense_variant	0.27
015-07	post-transplant	ZNF318	chr6:g.43308158T>C	ENSP00000354964.2:p.Glu1193Gly	missense_variant	0.57
015-07	pre-transplant	DNMT3A	chr2:g.25470480C>T	ENSP00000264709.3:p.Gly332Arg	missense_variant	0.41
015-07	post-transplant	DNMT3A	chr2:g.25470480C>T	ENSP00000264709.3:p.Gly332Arg	missense_variant	0.24
015-07	pre-transplant	DNMT3A	chr2:g.25464478C>T	ENSP00000264709.3:p.Gly679Arg	missense_variant	0.2
015-10	pre-transplant	DNMT3A	chr2:g.25457243G>A	ENSP00000264709.3:p.Arg882Cys	missense_variant	0.29
015-11	post-transplant	IDH2	chr15:g.90645517G>C	ENSP00000331897.3:p.Arg36Gly	missense_variant	0.71
015-12	post-transplant	PPM1D	chr17:g.58740697del	ENSP00000306682.2:p.Phe534LeufsTer5	frameshift_variant	15
015-12	post-transplant	PPM1D	chr17:g.58740749C>T	ENSP00000306682.2:p.Arg552Ter	stop_gained	2.27
015-12	post-transplant	PPM1D	chr17:g.58740697del	ENSP00000306682.2:p.Phe534LeufsTer5	frameshift_variant	37.84
015-12	post-transplant	PPM1D	chr17:g.58740697del	ENSP00000306682.2:p.Phe534LeufsTer5	frameshift_variant	24.42
015-12	pre-transplant	PPM1D	chr17:g.58740697del	ENSP00000306682.2:p.Phe534LeufsTer5	frameshift_variant	9.29
015-12	pre-transplant	PPM1D	chr17:g.58740749C>T	ENSP00000306682.2:p.Arg552Ter	stop_gained	0.92
015-12	post-transplant	PPM1D	chr17:g.58740749C>T	ENSP00000306682.2:p.Arg552Ter	stop_gained	0.75
015-12	post-transplant	PPM1D	chr17:g.58740749C>T	ENSP00000306682.2:p.Arg552Ter	stop_gained	1.16
015-13	pre-transplant	DNMT3A	chr2:g.25471058C>A	ENSP00000264709.3:p.Glu235Ter	stop_gained	2.02
015-16	post-transplant	ZNF318	chr6:g.43310556T>C	ENSP00000354964.2:p.Gln1045Arg	missense_variant	0.91
015-16	post-transplant	TP53	chr17:g.7577120_7577122dup	ENSP00000269305.4:p.Arg273dup	inframe_insertion	0.48
015-16	post-transplant	ASXL1	chr20:g.31021518_31021519del	ENSP00000364839.4:p.Arg506AsnfsTer3	frameshift_variant	0.77
015-16	pre-transplant	ASXL1	chr20:g.31021518_31021519del	ENSP00000364839.4:p.Arg506AsnfsTer3	frameshift_variant	0.39
015-16	pre-transplant	ASXL1	chr20:g.31023159del	ENSP00000364839.4:p.Gln882LysfsTer4	frameshift_variant	0.36
015-16	post-transplant	TFR2	chr7:g.100218647G>A	ENSP00000420525.1:p.His747Tyr	missense_variant	0.26
015-16	post-transplant	ASXL1	chr20:g.31021518_31021519del	ENSP00000364839.4:p.Arg506AsnfsTer3	frameshift_variant	0.76
015-16	post-transplant	ASXL1	chr20:g.31023159del	ENSP00000364839.4:p.Gln882LysfsTer4	frameshift_variant	0.31
015-17	post-transplant	SH2B3	chr12:g.111856239_111856260del	ENSP00000345492.2:p.Pro97ArgfsTer93	frameshift_variant	15.58
015-17	pre-transplant	SH2B3	chr12:g.111856239_111856260del	ENSP00000345492.2:p.Pro97ArgfsTer93	frameshift_variant	20.03
015-17	post-transplant	SH2B3	chr12:g.111856239_111856260del	ENSP00000345492.2:p.Pro97ArgfsTer93	frameshift_variant	16.05
015-19	post-transplant	CREBBP	chr16:g.3779024_3779032del	ENSP00000262367.5:p.Asn2006_Val2008del	inframe_deletion	0.2

Table S2: CH Variant details (continued)

UPN	Cohort	Gene	HGVSg	HGVSp	Consequence	VAF (%)
015-19	pre-transplant	IDH2	chr15:g.90630433A>G	ENSP00000331897.3:p.Met293Thr	missense_variant	0.47
015-19	post-transplant	IDH2	chr15:g.90630433A>G	ENSP00000331897.3:p.Met293Thr	missense_variant	0.16
015-24	pre-transplant	DNMT3A	chr2:g.25505449del	ENSP00000264709.3:p.Ser105AlafsTer57	frameshift_variant	0.76
015-24	post-transplant	PPM1D	chr17:g.58740809C>T	ENSP00000306682.2:p.Arg572Ter	stop_gained	2.77
015-24	pre-transplant	PPM1D	chr17:g.58740809C>T	ENSP00000306682.2:p.Arg572Ter	stop_gained	1.29
015-24	post-transplant	DNMT3A	chr2:g.25505449del	ENSP00000264709.3:p.Ser105AlafsTer57	frameshift_variant	0.23
015-25	post-transplant	PPM1D	chr17:g.58740809C>T	ENSP00000306682.2:p.Arg572Ter	stop_gained	1.33
015-25	pre-transplant	ATRX	chrX:g.76764032T>C	ENSP00000362441.4:p.Met2426Val	missense_variant	0.64
015-25	pre-transplant	PPM1D	chr17:g.58740809C>T	ENSP00000306682.2:p.Arg572Ter	stop_gained	1.12
015-26	post-transplant	SETBP1	chr18:g.42618495G>A	ENSP00000282030.5:p.Ser1349Asn	missense_variant	1.32
015-32	pre-transplant	DNMT3A	chr2:g.25468136del	ENSP00000264709.3:p.Cys514AlafsTer137	frameshift_variant	2.88
015-32	post-transplant	TP53	chr17:g.7578394T>C	ENSP00000269305.4:p.His179Arg	missense_variant	0.9
015-32	pre-transplant	TP53	chr17:g.7578394T>C	ENSP00000269305.4:p.His179Arg	missense_variant	0.75
015-32	post-transplant	DNMT3A	chr2:g.25468136del	ENSP00000264709.3:p.Cys514AlafsTer137	frameshift_variant	0.5
015-33	pre-transplant	TET2	chr4:g.106155891del	ENSP00000442788.1:p.Thr265LeufsTer28	frameshift_variant	1.48
12022	healthy	DNMT3A	chr2:g.25463563_25463575del	ENSP00000264709.3:p.Leu703AlafsTer72	frameshift_variant	0.64
12022	healthy	DNMT3A	chr2:g.25466800G>A	ENSP00000264709.3:p.Arg635Trp	missense_variant	1.23
12022	healthy	DNMT3A	chr2:g.25470498G>A	ENSP00000264709.3:p.Arg326Cys	missense_variant	0.64
12022	healthy	DNMT3A	chr2:g.25470532C>T	ENSP00000264709.3:p.Trp314Ter	stop_gained	0.79
12022	healthy	DNMT3A	chr2:g.25470575A>T	ENSP00000264709.3:p.Leu300Gln	missense_variant	0.69
12022	healthy	ZNF318	chr6:g.43336727_43336764del	ENSP00000354964.2:p.Ala116ArgfsTer14	frameshift_variant	1.34
12022	healthy	SRCAP	chr16:g.30733579del	ENSP00000262518.4:p.Ala1227LeufsTer77	frameshift_variant	0.46
12225	healthy	DNMT3A	chr2:g.25457242C>T	ENSP00000264709.3:p.Arg882His	missense_variant	1.18
12225	healthy	DNMT3A	chr2:g.25470532C>T	ENSP00000264709.3:p.Trp314Ter	stop_gained	0.53
12225	healthy	BRCC3	chrX:g.154305470T>A	ENSP00000358474.1:p.Ile74Asn	missense_variant	7.5
15189	healthy	DNMT3A	chr2:g.25463291del	ENSP00000264709.3:p.Tyr735ThrfsTer44	frameshift_variant	10.56
16780	healthy	DNMT3A	chr2:g.25467125del	ENSP00000264709.3:p.Tyr584ThrfsTer67	frameshift_variant	0.72
22996	healthy	ZNF318	chr6:g.43333109del	ENSP00000354964.2:p.Phe157SerfsTer30	frameshift_variant	1.22
26710	healthy	SH2B3	chr12:g.111885238C>T	ENSP00000345492.2:p.Gln376Ter	stop_gained	1.54
26710	healthy	ASXL1	chr20:g.31022415_31022437del	ENSP00000364839.4:p.Glu635ArgfsTer15	frameshift_variant	1.38
45268	healthy	DNMT3A	chr2:g.25463179del	ENSP00000264709.3:p.Leu773SerfsTer6	frameshift_variant	1.02
45268	healthy	DNMT3A	chr2:g.25463587C>T	ENSP00000264709.3:p.Gly699Ser	missense_variant	2.61
45268	healthy	DNMT3A	chr2:g.25470498G>A	ENSP00000264709.3:p.Arg326Cys	missense_variant	0.53
47517	healthy	CUX1	chr7:g.101844678A>G	ENSP00000353401.3:p.Asn712Asp	missense_variant	0.48
57745	healthy	DNMT3A	chr2:g.25463229A>G	ENSP00000264709.3:p.Phe755Ser	missense_variant	2.39
57745	healthy	BRCC3	chrX:g.154305494G>A	ENSP00000358474.1:p.Arg82His	missense_variant	1.11
61170	healthy	DNMT3A	chr2:g.25463536C>T	ENSP00000264709.3:p.Val716Ile	missense_variant	1.18
61170	healthy	ZNF318	chr6:g.43322770G>A	ENSP00000354964.2:p.Gln768Ter	stop_gained	0.64
61170	healthy	ZNF318	chr6:g.43323138del	ENSP00000354964.2:p.Cys645PhefsTer32	frameshift_variant	0.7

Table S2: CH Variant details (continued)

UPN	Cohort	Gene	HGVSg	HGVSp	Consequence	VAF (%)
63781	healthy	SMARCD2	chr17:g.61914909C>T	ENSP00000392617.2:p.Gly98Asp	missense_variant	1.99
63781	healthy	GNAS	chr20:g.57429095G>A	ENSP00000360141.3:p.Ala259Thr	missense_variant	1.29
65530	healthy	DNMT3A	chr2:g.25463508C>T	NA	splice_donor_variant	1.72
68786	healthy	TET2	chr4:g.106164913C>T	ENSP00000442788.1:p.Arg1261Cys	missense_variant	10.91
70161	healthy	RUNX1	chr21:g.36231773C>T	ENSP00000300305.3:p.Arg204Gln	missense_variant&splice_region_variant	1.77
73088	healthy	ZBTB33	chrX:g.119388636G>T	ENSP00000314153.2:p.Glu456Ter	stop_gained	1.04
76283	healthy	CUX1	chr7:g.101870762G>C	ENSP00000353401.3:p.Glu1093Asp	missense_variant	0.33
78691	healthy	DNMT3A	chr2:g.25458643_25458644insA	ENSP00000264709.3:p.Lys844Ter	frameshift_variant	1.65
78691	healthy	DNMT3A	chr2:g.25463291del	ENSP00000264709.3:p.Tyr735ThrfsTer44	frameshift_variant	0.58
87327	healthy	ASXL1	chr20:g.31024314_31024323delinsT	ENSP00000364839.4:p.Thr1267_Arg1270delinsTer	stop_gained	5.59
90221	healthy	DNMT3A	chr2:g.25469028C>T	NA	splice_donor_variant	0.51
90221	healthy	ASXL1	chr20:g.31022581C>G	ENSP00000364839.4:p.Ser689Ter	stop_gained	1.52
90221	healthy	ASXL1	chr20:g.31022922C>T	ENSP00000364839.4:p.Gln803Ter	stop_gained	1.45
90221	healthy	IL17RA	chr22:g.17589215A>G	ENSP00000320936.6:p.Asp369Gly	missense_variant	0.77
99830	healthy	SETD2	chr3:g.47103677del	ENSP00000386759.3:p.Gly2091GlufsTer56	frameshift_variant	2.37

UPN unique patient number; HGVS Human Genome Variant Society; VAF variant allele frequency

Table S3: Lung Transplant Recipient Clinical Characteristics by CH status

	No CH (N=38)	Non-DDR CH (N=23)	DDR CH (N=24)
Recipient Age, years, median (IQR)	55 (42-62)	62 (54-65)	66 (62-69)
Recipient Female Sex, N(%)	14 (34.1)	13 (56.5)	10 (41.7)
Recipient Race*, N(%)			
White	38 (92.7)	19 (82.6)	23 (95.8)
Non-White	3 (7.3)	4 (17.4)	1 (4.2)
Transplant Diagnosis, N(%)			
COPD	8 (21.1)	10 (43.5)	7 (29.2)
ILD	18 (47.4)	10 (43.5)	16 (66.7)
Other**	12 (31.6)	3 (13)	1 (4.2)
Pre-Tx Cigarette Use, N(%)	22 (53.7)	14 (60.9)	19 (79.2)
Pre-Tx Immunosuppression†, N(%)	11 (27.5)	7 (31.8)	9 (37.5)
Mycophenolate	6 (14.6)	4 (17.4)	2 (8.3)
Azathioprine	0 (0)	1 (4.3)	1 (4.2)
Methotrexate	2 (4.9)	0 (0)	0 (0)
Rituximab	1 (2.4)	1 (4.3)	0 (0)
Chronic Steroids	8 (19.5)	6 (26.1)	8 (33.3)
Recipient CMV Positive, N(%)	21 (55.3)	10 (43.5)	16 (66.7)
Transplant Type, N(%)			
Single	1 (2.4)	1 (4.3)	2 (8.3)
Bilateral	40 (97.6)	22 (95.7)	22 (91.7)
Ischemic Time, minutes, median (IQR)	272 (227-335)	290 (241-321)	282 (221-346)
Induction Therapy, (N(%)			
Basiliximab	26 (81.3)	15 (65.2)	21 (87.5)
ATGAM	4 (12.5)	1 (4.3)	0 (0)
Thymoglobulin	2 (6.3)	7 (30.4)	3 (12.5)
Anti-Proliferative Agent, N(%)			
MPA Tolerant	26 (63.4)	18 (78.3)	10 (41.7)
MPA Intolerant	15 (36.6)	5 (21.7)	14 (58.3)
Maintenance Calcineurin Inhibitor, N(%)			
Tacrolimus	41 (100)	23 (100)	23 (95.8)
Cyclosporine	0 (0)	0 (0)	0 (0)
None	0 (0)	0 (0)	1 (4.2)

COPD: Chronic obstructive lung disease, ILD: interstitial lung disease, MPA: mycophenolic acid

*Non-white: 7 Black/African American, 1 Asian. **Other: 7 cystic fibrosis, 3 Alpha-1 antitrypsin, 3 pulmonary hypertension, 1 sarcoid, 1 non-CF bronchiectasis, 1 Other

†Totals for each agent exceed total number of patients as many patients were on multiple immunosuppression agents simultaneously

Table S4: Genes Reviewed on Whole Exome Sequencing

ABCA3	*ACD*	*ANKRD26*	*AP3B1*	*ATG2B*	*ATM*
ATR	*BLM*	*BRCA1*	*BRCA2*	*BRIP1*	*CBL*
CEBPA	*CFTR*	*CHEK2*	*COPA*	*CTC1*	*DDX41*
DKC1	*DNAJC21*	*EFL1*	*ELANE*	*EPCAM*	*ERCC6L2*
ETV6	*FAM111B*	*FANCA*	*FANCB*	*FANCC*	*FANCD2*
FANCE	*FANCF*	*FANCG*	*FANCI*	*FANCL*	*FANCM*
G6PC3	*GATA1*	*GATA2*	*GBA*	*GFI1*	*GSKIP*
HAX1	*HPS1*	*HPS4*	*KRAS*	*LIG4*	*MAD2L2*
MARS1	*MBD4*	*MLH1*	*MLH2*	*MSH6*	*NAF1*
NF1	*NHP2*	*NKX2-1*	*NOP10*	*PALB2*	*PARN*
PMS2	*POT1*	*PPM1D*	*PTPN11*	*RAD51C*	*RNF168*
RPA1	*RPL11*	*RPL15*	*RPL23*	*RPL26*	*RPL27*
RPL31	*RPL35A*	*RPL5*	*RPS10*	*RPS24*	*RPS26*
RPS27	*RPS28*	*RPS29*	*RPS7*	*RTEL1*	*RUNX1*
SAMD9	*SAMD9L*	*SBDS*	*SFTPA1*	*SFTPA2*	*SFTPC*
SMPD1	*SRP72*	*STING1*	*STN1*	*TEN1*	*TERF1*
TERF2	*TERF2IP*	*TERT*	*TINF2*	*TP53*	*TSR2*
UBE2T	*USB1*	*WRAP53*	*XPC*	*XRCC2*	*ZCCHC8*
rs35705950 (MUC5B promoter)					

Table S5: Germline Variants with Pathogenic or Uncertain Significance

ID	Diagnosis	Age	Gene	Variant
013-65	ILD	48	RTEL1	ENST00000370018.7:exon24:c.G2105A:p.Arg702His
			TERT	ENST00000310581.9:exon5:c.C2092T:p.Arg698Trp
008-57	HP	56	RTEL1	ENST00000370018.7:exon14:c.C1189G:p.Gln397Glu
015-03	ILD	60	RTEL1	ENST00000370018.7:exon23:c.T1955C:p.Met652Thr
013-59	ILD	61	PARN	ENST00000341484.11:exon5:c.A89G:p.Tyr30Cys
009-04	ILD	65	TERT	ENST00000310581.9:exon5:c.G2130T:p.Lys710Asn
008-40	COPD	65	TERT	ENST00000310581.9:exon4:c.C1880G:p.Pro627Arg
015-15	HP	64	RTEL1	ENST00000370018.7:exon30:c.G2903C:p.Cys968Ser
015-34	ILD	75	RTEL1	ENST00000370018.7:exon7:c.A540T:p.Glu180Asp
010-25	ILD	68	TERT	ENST00000310581.9:exon2:c.C1396T:p.Arg466Trp
010-20	ILD	59	PARN	ENST00000539279.5:exon10:c.A626G:p.Tyr209Cys
015-25	ILD	63	SFTPC	ENST00000522109.5:exon3:c.G299T:p.Gly100Val
009-08	ILD	43	SFTPA2	ENST00000372327.9:exon5:c.G633T:p.Trp211Cys
006-26	ILD	49	SFTPA1	ENST00000428376.6:exon5:c.G725A:p.Arg242Gln
008-34	ILD, MDS	68	DDX41	ENST00000625286.1:exon1:c.G3A:p.Met1Ile
